# Assessment of ventilatory functions and associated inflammatory markers among workers in slaughterhouses

**DOI:** 10.1007/s00420-024-02094-8

**Published:** 2024-08-16

**Authors:** Mona Abdallah Ramadan, Rateba said Mohammed, Aisha Safwat Saif Eldin

**Affiliations:** 1 Department of Occupational and Environmental Medicine, Faculty of Medicine, Cairo University, Giza, Egypt; 2 College of Health and Rehabilitation Sciences, Princess Nora Bint Abul Rahman University, Riyadh, Saudi Arabia

**Keywords:** Slaughterhouse, Respiratory hazards, Ventilatory function parameters, IL-6, hsCRP

## Abstract

**Objective:**

Meat processing is among the most extensive industries globally. However, data on the effects of occupational exposure on the pulmonary health of slaughterhouse workers is limited. Ascertaining the impact of the slaughterhouse atmosphere on the breathing habits of laborers exposed to it and the inflammatory markers associated with it was the aim of the current investigation.

**Methods:**

A cross-sectional study was performed on 82 non-smoker subjects of 41 male workers working in one of the major slaughterhouses in Cairo, Egypt, matched to 41 controls of administrative personnel. An elaborate questionnaire encompassing medical and occupational history was administered to each participant in the research. General and local systemic examinations and ventilatory function tests were carried out, and serum levels of interleukin 6 (IL-6) and high-sensitivity C-reactive protein (hsCRP) were measured.

**Results:**

Respiratory symptoms were more prevalent with a statistically significant decline in ventilatory function parameters (FVC%, FEV1%, FEV1/FVC, FEF 25%, FEF 50%, FEF 75%, and PEF%) among the exposed group compared to those of control. In addition, there was a significantly higher serum level of inflammatory markers (IL-6 and hsCRP) among the exposed group compared to the control group, with a negative correlation with ventilatory functions. Moreover, there was a positive association between levels of serum IL-6 and hsCRP and the age and duration of employment of workers.

**Conclusion:**

There was a notable increase in the prevalence of respiratory disorders and inflammatory markers among slaughterhouse workers. Additionally, there was a substantial decrease in ventilatory function parameters, which could be attributed to the bioaerosols they encountered in the workplace.

**Supplementary Information:**

The online version contains supplementary material available at 10.1007/s00420-024-02094-8.

## Introduction

The meat processing sector is among the most extensive industries globally, employs a sizable workforce, and is vital to most urban areas’ economic and social health (Swai and Schoonman 2012). Working in a slaughterhouse may be linked to health risks that lead to occupational illnesses or exacerbate preexisting health issues of non-occupational origin (WHO 2001). Various types of labor expose a significant number of slaughterhouse employees to perilous circumstances daily. However, occupational hazards account for most illnesses and fatalities among all laborers (Abdullahi et al. 2016).

The staff of slaughterhouses are confronted with a diverse range of biological pollutants, such as fungi and bacteria, due to the working environment. The contaminants above, known as “bioaerosols,” can be created and dispersed in the workplace during various slaughtering operations, such as shaving the head, cleaning the rumen, and cleaning the colon. Employees who come into contact with these contaminants may experience immunological responses that are believed to exacerbate breathing issues and subsequent complications (Martenies et al. 2020).

Significant levels of bioaerosols have been identified as the cause of organic dust toxic conditions, a severe, non-allergic illness exhibiting features comparable to influenza (Madsen et al. 2012). One of the principal adverse health effects of bioaerosols is mucous membrane irritation in the upper airways and eyes, which does not involve an allergic reaction (Pearson et al. 2015). There exists a correlation between prolonged exposure to bioaerosols and the progression of chronic bronchitis, chronic obstructive pulmonary disease (COPD), and impaired pulmonary function (van Kampen et al. 2016). Lower respiratory tract irritation signs, such as congestion, declines in lung function, and phlegm, have been documented in individuals employed in composting facilities and poultry farms (Hambach et al. 2012; Viegas et al. 2013).

During the initial phases of inflammation, the soluble inflammatory factor IL-6 is produced. However, this is accompanied by the rapid activation of numerous acute-phase proteins, such as fibrinogen, serum amyloid A (SAA), and CRP (Tanaka et al. 2014). Maintenance and induction of chronic inflammation are primarily mediated by IL-6. There is accumulating evidence linking amounts of IL6 to COPD. There exists an inverse correlation between increased amounts of IL-6 and lung capacity metrics, including forced expiratory volume in one second as a percentage of expected value (FEV1% pred), which are both of which are observed among individuals with COPD (Grubek-Jaworska et al. 2012; Huang et al. 2021).

Blood samples containing elevated concentrations of acute-phase response proteins may function as prophylactic indicators of adverse health consequences. Consequently, acute-phase CRP concentrations in the blood lead to an increase in response to inflammation, infection, or tissue injury (Rasmussen et al. 2023). Airflow restriction and airway inflammation are additionally associated with elevated levels of hsCRP (Takemura et al. 2006).

The impact of bioaerosol exposure on the respiratory health of slaughterhouse employees has only been investigated in a few research studies. The goal of this study was to compare slaughterhouse workers to healthy office workers, determine if exposure to bioaerosols in the workplace was associated with changes in ventilatory function tests evaluated by post-work shift spirometry, and investigate the plasma levels of IL-6 and hsCRP as primary inflammatory triggers.

## Materials and methods

### Study design and population

A cross-sectional investigation was performed in one of the major slaughterhouses in Greater Cairo, Egypt, from August 2023 to December 2023. Forty-one male employees of the industrial slaughterhouse (cows and sheep) participated in this investigation. The present investigation randomly selected 41 healthy office workers (males) from the same industry who shared similar socioeconomic and demographic characteristics (e.g., education, smoking behaviors, and ethnic background) and had no prior exposure to pollutants recognized for their potential to induce respiratory conditions.

### Sample size calculation

Based on data from Kasaeinasab et al. (2017) and comparing pulmonary function tests (VC) between exposed and non-exposed workers, 41 exposed and 41 non-exposed workers were needed for this study. Using the G*power program, an independent sample T-test family with effect size dz was used. However, 0.76 at 90% power and a 0.05 significant level were applied. Meanwhile, the dropout rate is 10%.

The Ethical Committee of the Faculty of Medicine, Kasr Al-Ainy Hospital, Cairo University, Egypt, granted sanction for this research before its implementation (N-483-2023). The study was executed in adherence to the ethical principles outlined in the Declaration of Helsinki in October 2013. After elucidating the purpose and significance of the research, informed consent was voluntarily obtained from all participants. Strict confidentiality was maintained throughout the process, including gathering samples, testing, coding, and recording results. Individuals may be granted copies of the serological test results so that they may proceed with subsequent investigations and treatment.

### Questionnaire and clinical examination

A comprehensive explanation was provided to the employees regarding the examination procedures and research plan. The reference group and employees from various sections of the industrial slaughterhouses were interviewed, and the researchers collected data through a questionnaire that inquired about personal, occupational, and workplace exposure, in addition to current and previous family history. Additionally, each participant underwent a comprehensive clinical examination. An investigation was conducted into the prevalence of respiratory symptoms, including wheezing, productive cough, cough, breathlessness, and sputum, using the respiratory symptoms questionnaire recommended by the American Thoracic Society (ATS). Moreover, weight and height were assessed. Weight in kilograms divided by height in square meters yielded the body mass index (BMI).

### Inclusion criteria

Individuals who were employed for a minimum of one year, did not smoke, and had no prior medical history pertaining to respiratory conditions were eligible to participate in the research.

### Exclusion criteria

Individuals who declined to participate in the research were those who were smokers, had a personal or familial medical history of respiratory conditions, had undergone chest surgery, were experiencing fever, had hepatitis, had an autoimmune disorder, or had any other current symptoms or indications of inflammation that could lead to an increase in hsCRP levels.

### Ventilatory function tests

A portable spirometry device called the “Flow Handy ZAN 100 USB Pulmonary Spirometer” (Betterflow), manufactured by ZAN Messgerate GmbH, Oberthulba, Germany, was used to evaluate ventilatory function parameters. The test was carried out on the scene in an adjacent administrative office. Before performing their pulmonary function tests, all the subjects were briefed on the process. Everybody was sitting upright and comfortably. To do the tests, the research participants had to put on a nasal clip and insert the mouthpiece and transducer. The mouthpiece was single use for every study participant. Enough support was given to the participants to perform deep, rapid inspiration and deep, rapid expiration.

Several parameters were measured with a single expiratory effort. After three repetitions of the examination, the best score obtained was documented. The normal predicted values were determined by providing the ATS/ERS guidelines regarding the height, gender, weight, age, and BMI of the participants (Miller et al. 2005). The severity of obstruction was assessed using the GOLD classification (Agustí et al. 2023). Obstruction is diagnosed when the FEV1/FVC ratio is less than 70% of the predicted value. A restrictive pattern is characterized by a reduction in the FVC ratio below 80% of the predicted value.

### Blood collection and sample preparation

Before the shiftwork, a venipuncture was performed in the early morning, between 7 and 8 am, to obtain 5 mL of blood from each subject’s arm under aseptic conditions, using tubes devoid of any additives or anticoagulants. The subjects were fasting and sitting comfortably upright during the procedure. Within two hours, the samples were sent to the laboratory on ice. After that, they were allowed to clot before being centrifuged at 7930 x g for 10 min using a Sigma laboratory centrifuge (3K30). The serum was then analyzed for hsCRP and IL-6 presence.

### Measurement of IL-6 and hsCRP

The hsCRP and IL-6 levels in the serum samples were determined using a quantitative double-antibody sandwich enzyme-linked immunosorbent assay (ELISA) method. Kits for this purpose were readily available from the manufacturer, and the procedure was carried out as directed. The calculations were performed following the standard curve. IL6 (ENZO, USA, Catalog #: ENZ-KIT178), hsCRP (BOSTER, CA, USA, Catalog number #: EK7040).

### Statistical analysis

The Statistical Package for the Social Sciences (SPSS) version 28 (IBM Corp., Armonk, NY, USA) was utilized to code and input the data. For quantitative variables, the data were summarized using the mean and standard deviation; for categorical variables, the frequencies (number of cases) and relative frequencies (percentages) were utilized. Group comparisons were assessed utilizing an unpaired t-test. The degrees of obstruction and restriction were compared using non-parametric Mann-Whitney and Kruskal-Wallis tests with multiple comparisons post hoc test (Chan 2003a). The chi-square (χ^2^) was employed to compare categorical data. Conversely, when the expected frequency was below five, the exact test was employed (Chan 2003b). The Pearson correlation coefficient was utilized to associate quantitative variables (Chan 2003c). Linear regression analysis was done to predict FVC, FEV1, and FEV/FVC (Chan 2004). To identify the optimal cut-off value of IL6 and hsCRP for case detection, an area under the curve analysis was used to construct a ROC curve. The criterion for statistical significance was a P-value below 0.05.

## Results

The current investigation involved 82 non-smoking participants, comprising 41 male workers from a prominent slaughterhouse in Cairo, Egypt. These were matched based on age, sex, and body mass index with 41 control individuals from administrative staff. Most of them wore personal protective equipment, including gloves and boots, but none wore respiratory masks. No statistically significant differences were observed between the cases and controls regarding age, duration of employment, and BMI (Table 1).

The results of the ventilatory function test showed a statistically significant decrease in the parameters of ventilatory functions such as FVC%, FEV1%, FEV1/FVC, FEF 25%, FEF 50%, FEF 75%, and PEF% among the exposed group compared to the control. Regarding clinical manifestations, a statistically significant elevation in systolic blood pressure was observed in the exposed group compared to the control group (133.9 ± 19.48 versus 126.59 ± 10.87, respectively) (Table 1).

The exposed group exhibited a higher prevalence of respiratory symptoms, including sinusitis, persistent cough, phlegm, wheezes, and breathlessness, compared to the control group, with a statistically significant difference (p-value < 0.001). In addition, when comparing the exposed group to the control group, 31.7% exhibited a moderate degree of obstruction, compared to 7.3% in the control group. Additionally, 17.1% showed severe obstruction, and 4.9% displayed very severe obstruction, while none in the control group had either severe or very severe obstruction. These differences were statistically significant. No statistically significant difference was observed between the two studied groups regarding the degree of severity of the restriction (Table 2).

Furthermore, the exposed group exhibited higher serum levels of inflammatory markers IL-6 compared to the control group (136.85 ± 58.47 versus 74.67 ± 28.16, respectively), as well as higher levels of hsCRP (5.28 ± 2.02 versus 3.54 ± 1.42, respectively) (Table 1).

The degree of obstruction severity among the exposed group was compared to their BMI, age, duration of employment, and serum levels of inflammatory markers. A higher degree of airway obstruction was associated with an increase in age and duration of employment. Statistically significant increases in IL-6 and hsCRP were additionally associated with higher degrees of obstruction severity (Table 3).

There was a statistically significant positive correlation between serum IL-6 and age, duration of employment, degrees of obstruction, degrees of restriction, and hsCRP. A negative correlation was found between serum IL-6 and FVC%, FEV1%, FEV1/FVC, FEF25%, FEF50%, FEF75%, and PEF% among the exposed group. Furthermore, a statistically significant positive association was found between serum hsCRP levels in the exposed group and age, duration of employment, and degrees of obstruction. Additionally, a negative correlation was noted between serum hsCRP levels in the exposed group and FEV1%, FEV1/FVC, FEF25%, FEF50%, FEF75%, and PEF% (Table 4).

Multivariate linear regression analysis confirmed a negative relationship between FVC, FEV/FVC, FEV1, and serum levels of IL6. (Table 5).

**Fig. 1:**
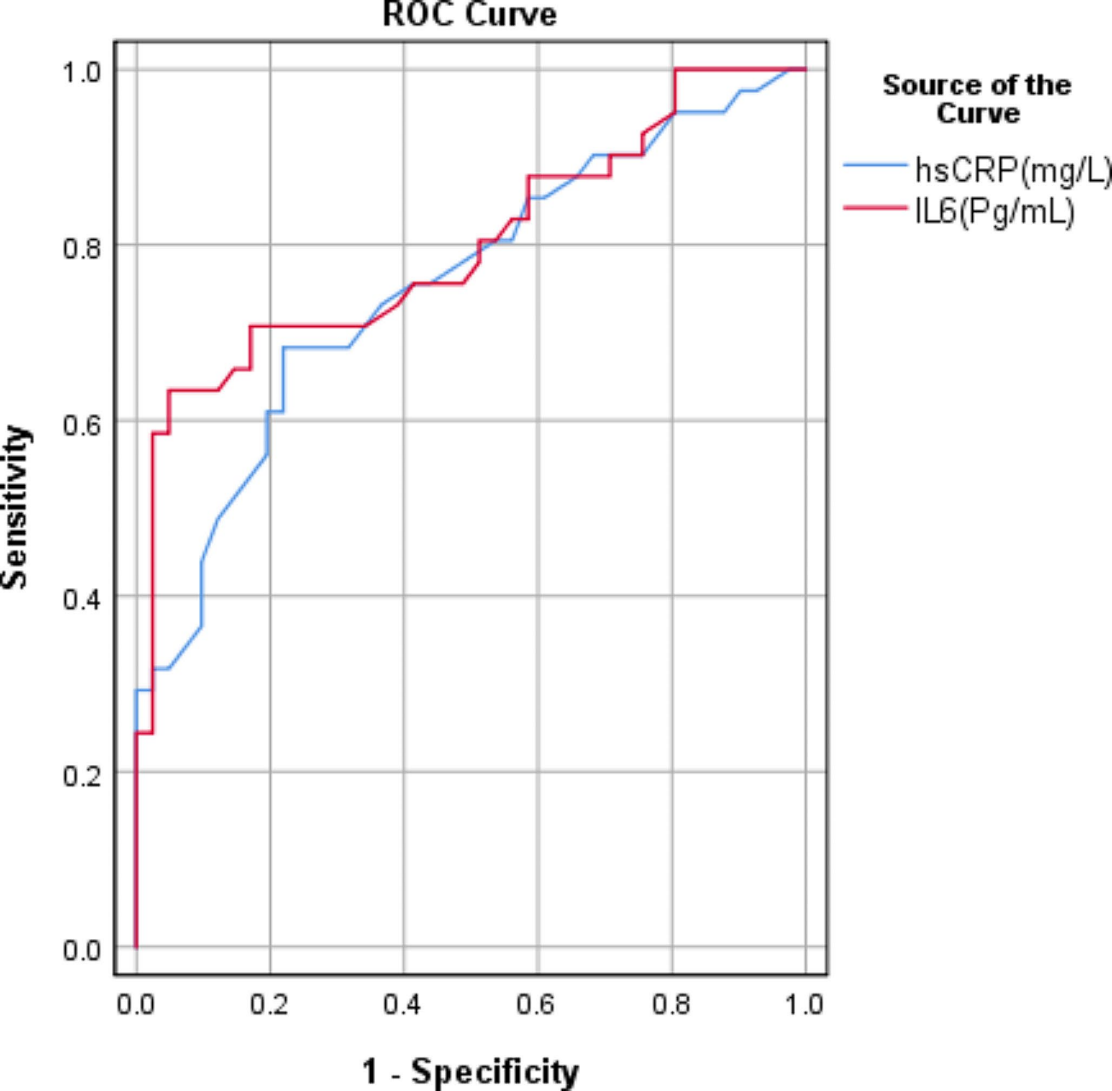
The figure shows ROC curve analysis for serum inflammatory markers IL-6 and hsCRP as predictors for obstructive ventilatory impairment among slaughterhouse workers

A ROC curve analysis (Fig. 1) was conducted for serum inflammatory markers IL-6 and hsCRP as predictors for obstructive ventilatory impairment among slaughterhouse workers. The area under the curve for IL-6 and hsCRP was notably higher, with sensitivity and specificity values of 63.4% and 95.1% for IL-6 and 68.3% and 78% for hsCRP, respectively. Optimal cut-off values of 115.95 pg/mL for IL-6 and 4.35 mg/L for hsCRP were determined.

**Table 1:** Demographic characteristics, anthropometric, ventilatory function parameters, and serum levels of inflammatory markers among the studied subjects

	Exposed group				Control				
	Mean	Standard deviation	Minimum	Maximum	Mean	Standard deviation	Minimum	Maximum	*p*-value
**Age**	45.22	11.53	27.00	60.00	45.44	10.62	30.00	60.00	0.929
**Duration of employment (years)**	22.83	12.34	6.00	42.00	23.71	11.79	6.00	40.00	0.743
**BMI**	27.08	5.87	17.58	42.20	27.30	6.12	17.00	41.00	0.867
**Systolic blood pressure**	133.90	19.48	100.00	170.00	126.59	10.87	100.00	140.00	0.040^*^
**Diastolic blood pressure**	85.85	12.24	60.00	120.00	81.59	7.78	70.00	100.00	0.064
**FVC%**	75.32	14.62	36.00	102.00	82.10	13.54	50.00	107.00	0.032^*^
**FEV1%**	64.26	19.66	20.00	103.00	77.34	15.55	45.00	106.00	0.001^*^
**FEV1/FVC**	69.71	13.71	29.70	92.70	84.23	14.86	55.00	122.00	< 0.001^*^
**FEF25%**	54.62	26.24	16.00	112.00	67.51	18.96	35.00	109.00	0.013^*^
**FEF50%**	49.96	26.86	12.00	112.00	66.95	16.51	33.00	98.00	0.001^*^
**FEF75%**	41.36	22.72	8.00	99.00	61.76	17.26	19.00	104.00	< 0.001^*^
**PEF%**	40.36	19.13	12.00	91.00	60.83	17.11	22.00	98.00	< 0.001^*^
**IL6 (pg/mL) **	136.85	58.47	49.70	252.30	74.67	28.16	36.80	185.40	< 0.001^*^
**hsCRP (mg/L)**	5.28	2.02	1.20	8.60	3.54	1.42	1.10	6.90	< 0.001^*^

BMI: body mass index; FVC: forced vital capacity; FEV1: forced expiratory volume in the first second; FEF: forced expiratory flow; PEF: peak expiratory flow; IL-6: interleukin 6; hsCRP: high-sensitivity C reactive protein

*p-value < 0.05 denotes statistical significance

**Table 2:** Distribution of clinical manifestation, degree of restriction, and degree of obstruction among the studied subjects

		Exposed group		Control		p-value
		Count	%	Count	%	
**Nasal irritation**	**Yes**	5	12.2%	1	2.4%	0.201
	**No**	36	87.8%	40	97.6%	
**Rhinitis**	**Yes**	7	17.1%	1	2.4%	0.057
	**No**	34	82.9%	40	97.6%	
**Sinusitis**	**Yes**	9	22.0%	1	2.4%	0.007^*^
	**No**	32	78.0%	40	97.6%	
**Persistent cough**	**Yes**	19	46.3%	2	4.9%	< 0.001^*^
	**No**	22	53.7%	39	95.1%	
**Phlegm**	**Yes**	16	39.0%	2	4.9%	< 0.001^*^
	**No**	25	61.0%	39	95.1%	
**Hemoptysis**	**Yes**	0	0.0%	0	0.0%	-----
	**No**	41	100.0%	41	100.0%	
**Chest pain**	**Yes**	4	9.8%	2	4.9%	0.675
	**No**	37	90.2%	39	95.1%	
**Wheezes**	**Yes**	12	29.3%	0	0.0%	< 0.001^*^
	**No**	29	70.7%	40	100.0%	
**Breathlessness**	**Yes**	18	43.9%	5	12.2%	0.001^*^
	**No**	23	56.1%	36	87.8%	
**Degree of restriction**	**No restriction**	17	41.5%	25	61.0%	0.094
	**Mild restriction**	10	24.4%	11	26.8%	
	**Moderate restriction**	9	22.0%	2	4.9%	
	**Moderately severe**	4	9.8%	3	7.3%	
	**Severe**	1	2.4%	0	0.0%	
**Degree of obstruction**	**No obstruction**	19	46.3%	38	92.7%	< 0.001^*^
	**Mild obstruction**	0	0%	0	0%	
	**Moderate obstruction**	13	31.7%	3	7.3%	
	**Severe obstruction**	7	17.1%	0	0.0%	
	**Very severe obstruction**	2	4.9%	0	0.0%	

*p-value < 0.05 denotes statistical significance

**Table 3:** Comparing different degrees of obstruction with serum levels of inflammatory markers, BMI, age, and duration of employment among exposed subjects

	Degree of obstruction										
	No obstruction			Moderate obstruction				Severe and very severe obstruction		*p*-value	
	Mean		SD	Mean		SD		Mean	SD		
**IL-6 (pg/mL)**	83.99		33.28	168.74		17.32		202.38	32.15	< 0.001^*^	
**hsCRP (mg/L)**	3.75		1.43	6.02		1.15		7.43	1.45	< 0.001^*^	
**BMI**	28.84		6.64	24.92		5.16		26.36	3.65	0.189	
**Age**	39.74		11.61	48.38		11.47		52.22	4.58	0.031^*^	
**Duration of employment (years)**	16.84		12.59	26.92		11.84		29.56	5.55	0.038^*^	
	**Degree of restriction**										
	**No restriction**			**Mild restriction**			**Moderate restriction**		**Moderately severe and severe restriction**		**P-value**
	**Mean**	**Standard Deviation**		**Mean**	**Standard Deviation**		**Mean**	**Standard Deviation**	**Mean**	**Standard Deviation**	
**BMI**	25.54	5.10		29.88	7.52		26.96	5.77	26.97	4.18	0.484
**Age**	45.94	11.75		42.90	13.58		45.11	11.49	47.60	8.62	0.920
**Duration of employment (years)**	23.76	13.06		20.70	13.14		22.44	12.96	24.60	9.79	0.949
**IL6(pg/mL)**	115.49	52.64		141.02	52.02		152.00	60.67	173.86	73.78	0.144
**hsCRP(mg/L)**	4.93	1.56		5.26	1.89		5.36	2.49	6.34	2.94	0.478

BMI: body mass index; IL-6: interleukin 6; hsCRP: high-sensitivity C reactive protein; SD: standard deviation

*p-value < 0.05 denotes statistical significance

**Table 4:** Correlation between inflammatory markers and demographic, ventilatory, and function test parameters among the exposed group (N: 41)

		IL-6 (pg/mL)	hsCRP (mg/L)
**BMI**	**Pearson correlation**	− 0.289	− 0.297
	**p-value**	0.067	0.060
**Age**	**Pearson correlation**	0.593	0.593
	**p-value**	< 0.001^*^	< 0.001^*^
**Duration of employment (years)**	**Pearson correlation**	0.610	0.615
	**p-value**	< 0.001^*^	< 0.001^*^
**FVC%**	**Pearson correlation**	− 0.408	− 0.252
	**p-value**	0.008^*^	0.112
**FEV1%**	**Pearson correlation**	− 0.770	− 0.589
	**p-value**	< 0.001^*^	< 0.001^*^
**FEV1/FVC**	**Pearson correlation**	− 0.853	− 0.727
	**p-value**	< 0.001^*^	< 0.001^*^
**FEF25%**	**Pearson correlation**	− 0.684	− 0.476
	**p-value**	< 0.001^*^	< 0.001^*^
**FEF50%**	**Pearson correlation**	− 0.741	− 0.570^*^
	**p-value**	< 0.001^*^	< 0.001^*^
**FEF75%**	**Pearson correlation**	− 0.715	− 0.564
	**p-value**	< 0.001^*^	< 0.001^*^
**PEF%**	**Pearson correlation**	− 0.742	− 0.596
	**p-value**	< 0.001^*^	< 0.001^*^
**hsCRP (mg/L)**	**Pearson correlation**	0.877	
	**p-value**	< 0.001^*^	
**Degree of restriction **	**Pearson correlation**	0.353	0.206
	**p-value**	0.023^*^	0.195
**Degree of obstruction**	**Pearson correlation**	0.889	0.767
	**p-value**	< 0.001^*^	< 0.001^*^

BMI: body mass index; FVC: forced vital capacity; FEV1: forced expiratory volume in the first second; FEF: forced expiratory flow; PEF: peak expiratory flow; IL-6: interleukin 6; HsCRP: high-sensitivity C reactive protein

*p-value < 0.05 denotes statistical significance

**Table 5:** Regression analysis for prediction of FVC, FEV1, and FEV/FVC among exposed subjects

Model		Unstandardized coefficients		Standardized coefficients	t	p-value	95.0% confidence interval for B	
		B	Std. Error	Beta			Lower Bound	Upper Bound
**FVC**	**(Constant)**	89.277	5.430		16.440	< 0.001^*^	78.293	100.261
	**IL6 (pg/mL)**	− 0.102	0.037	− 0.408	− 2.789	0.008^*^	− 0.176	− 0.028
**FEV1**	**(Constant)**	110.589	20.321		5.442	< 0.001^*^	69.375	151.803
	**IL6(Pg/mL)**	-0.407	0.073	-1.211	-5.604	< 0.001^*^	-0.555	-0.260
	**hsCRP(mg/L)**	3.724	2.118	0.382	1.758	0.087	-0.571	8.019
	**Age**	-0.667	0.797	-0.391	-0.836	0.408	-2.284	0.950
	**years of employment**	0.873	0.760	0.548	1.149	0.258	-0.668	2.414
**FEV/FVC**	**(Constant)**	107.145	12.302		8.710	< 0.001^*^	82.196	132.095
	**IL6(pg/mL)**	-0.235	0.044	-1.000	-5.331	< 0.001^*^	-0.324	-0.145
	**hsCRP(mg/L)**	0.789	1.282	0.116	0.615	0.542	-1.811	3.389
	**Age**	-0.462	0.483	-0.389	-0.958	0.344	-1.441	0.516
	**years of employment**	0.500	0.460	0.450	1.087	0.284	-0.433	1.433

BMI: body mass index; FVC: forced vital capacity; FEV1: forced expiratory volume in the first second; IL-6: interleukin 6; hsCRP: high-sensitivity C reactive protein

*p-value < 0.05 denotes statistical significance

## Discussion

Slaughterhouse work is notorious for its harsh conditions and risks to slaughterhouse workers’ health. Among the numerous hazards they face is the high level of bioaerosols, which are underappreciated and largely invisible threats. These organic dust particles, including bacteria and fungi, pass through the air in such environments, posing serious threats to the respiratory health of workers. This study assessed lung function parameters, respiratory symptoms, and associated serum inflammatory markers among slaughterhouse workers.

The study’s results indicated that age and BMI did not differ significantly between the two categories. Nevertheless, an increase in the incidence of respiratory problems and a significant decrease in lung function measurements were identified among laborers in slaughterhouses. The incidence rates of sinusitis, persistent cough, phlegm, wheezes, and breathlessness among workers in slaughterhouses were 22%, 46.3%, 39%, 29.3%, and 43.9%, respectively. Further, these were significantly higher than those of the unexposed workers. These findings agree with those of Kasaeinasab et al. (2017), who worked with 81 slaughterhouse workers in Iran. However, they reported that exposure to bioaerosols was linked to a significant decrease in lung function metrics and an increase in the frequency of respiratory issues among slaughterhouse workers.

Regarding the incidence of respiratory issues and ventilatory function metrics among slaughterhouse employees, to the best of our knowledge, little research has been conducted; therefore, comparing the results is impossible. Nonetheless, the frequency of respiratory conditions among slaughterhouse workers brought on by bioaerosol exposure matched that of workers in poultry farms, composting plants, wastewater treatment, and solid waste management (Viegas et al. 2013; Jahangiri et al. 2015; Taluja et al. 2018).

This research revealed that 31.7% of individuals employed in slaughterhouses experienced moderate airway obstruction, 17.1% had severe airway obstruction, and 4.9% had extremely severe airway obstruction. The mean observed values of FEV1, FEV1/FVC, and FVC were 64.26 ± 19.66, 69.71 ± 13.71, and 75.32 ± 14.62, respectively. Comparatively, obstruction was associated with considerably reduced FVC/FEV1 values among personnel in other animal care facilities, such as poultry houses (Yasmeen et al. 2020).

The current work showed that a higher degree of obstruction was associated with an increase in age and duration of exposure, which is in line with a study conducted by Younis and his colleagues (2020), which suggested that poultry laborers’ years of experience were positively correlated with a decline in lung function capacity.

The results of this study indicated that exposed laborers exhibited a greater inflammatory response than the control group, as measured by increased hsCRP and IL-6 amounts. However, this could be attributed to a high-stress workplace that negatively impacts employee health and elevates oxidative stress, thereby precipitating a rise in inflammation. These results align with those of Zelzer et al. (2018), who examined inflammation and work intensity levels among office and abattoir workers.

Bioaerosol exposure doesn’t just impact the respiratory system directly; it triggers systemic inflammatory responses. Wang and his colleagues (1997) found that exposure to swine dust led to a significant increase in interleukin-6 in the blood. This increase in IL-6 suggests that exposure to bioaerosols triggers an immune response that can affect the entire body, not just the respiratory system.

IL6, which plays a crucial role in triggering and sustaining chronic inflammation, is a keystone cytokine in inflammation. It is synthesized in an area during the early stages of inflammation and subsequently migrates to the liver, where it rapidly induces a wide array of acute-phase proteins, including CRP, fibrinogen, and SAA (Tanaka et al. 2014).

Additionally, elevated levels of hsCRP and IL-6 were found to be correlated with more severe obstructions. This finding aligns with numerous studies reporting higher circulating serum levels of hsCRP and IL-6 among individuals with COPD compared to control subjects. Furthermore, these levels were observed to increase with disease progression. A role in the regulation of the acute inflammatory response, including the control of CRP synthesis and secretion, is played by elevated levels of these markers among individuals with COPD (Bolton et al. 2007; De Moraes et al. 2014; Hussein et al. 2022) .

Moreover, the present study revealed a significant inverse correlation between IL-6 and hsCRP levels and FEV1%, FEV1/FVC, FEF25%, FEF 50%, FEF 75%, and PEF%. However, this agrees with Hussein et al. (2022), who found a significant negative correlation between IL-6 levels and FEV1%, FVC%, and FEF25–75% predicted values.

It is possible to reconcile the correlation between the reduction in FEV1 and FEV1% and the degree of inflammation, fibrosis, and luminal exudates in the small airways. Furthermore, COPD sufferers experience airflow limitation due to increased airflow resistance, which is induced by smooth muscle hypertrophy, goblet cell metaplasia, airway cartilage loss, and mucosal hypersecretion (Haraguchi et al. 1999).

Prolonged exposure to occupational stressors has been found to reduce antioxidative capacity, potentially amplifying the adverse effects of heightened oxidative stressor production resulting from a substantial caseload (Zimet et al. 2016). Li et al. (2012), performed a systematic review of research examining the effects of persistent exposure to air pollution, particulate matter, and smoke on CRP levels in children and adults. The authors discovered that patients exposed for an extended period exhibited elevated CRP levels.

The present investigation revealed a statistically significant positive correlation between the amount of serum hsCRP and IL-6 in the exposed group and age and duration of employment, respectively. This result is comparable to that of Walker et al. (2016), who found inflammation and immune system changes linked to protracted occupational exposure; thus, it emphasizes the need for enhanced workplace risk management.

In addition, a ROC curve was performed for IL-6 and hsCRP as predictors of obstructive ventilatory impairment among slaughterhouse workers. The results indicated that IL-6 and hsCRP had a significantly greater area under the curves, with sensitivity and specificity of 63.4% and 95.1%, and 68.3% and 78%, respectively. The optimal cut-off values for IL-6 and hsCRP were 115.95 (pg/mL) and 4.35 (mg/L), respectively. Huang et al. (2021), hypothesized, based on the ROC curve and AUC, that IL-6 might more accurately reflect the level of systemic inflammation in COPD patients than hs-CRP.

Furthermore, an investigation conducted by Yang et al. (2022), utilized ROC analysis to identify serum hsCRP and IL-6 as autonomous risk factors for chronic obstructive pulmonary disease in conjunction with pulmonary hypertension. Sensitivity, specificity, and critical value for the serum concentration of hsCRP were 90.91%, 85.96%, and 38.49 (mg/L), respectively; sensitivity, specificity, and critical value for IL-6 were 87.27%, 89.47%, and 98.99 (pg/mL), respectively.

Finally, it is, thus, plausible that these cytokines, which are probably produced and released in the airways following exposure to bioaerosols in slaughterhouse environments, are important for the inflammatory reaction and, to some extent, may explain some of the symptoms following exposure.

## Conclusions and recommendations

The health hazards faced by slaughterhouse workers extend beyond the physical risks associated with handling animals and sharp instruments. In a typical slaughterhouse environment, the concentration of bioaerosols is alarmingly high due to the nature of the work, which includes killing and processing animals and the handling of animal waste. Slaughterhouse employees must be made aware of this concealed health risk, and efforts must be made to establish safer working conditions. In summary, the findings of this research demonstrated a rise in the incidence of respiratory symptoms and a decline in lung function outcomes among slaughterhouse employees. Additionally, the present study unveiled elevated concentrations of the inflammatory markers hsCRP and IL-6, which exhibited a negative association with respiratory functions but a positive correlation with age and length of employment among workers who were exposed. This correlation could be attributed to the bioaerosols in the workers’ workplace. Although some precautions can be taken to minimize exposure, it is difficult to completely avoid bioaerosols due to the inherent nature of slaughterhouse labor. Hence, continuous investigation is required to enhance comprehension of these hazards and formulate more efficacious preventive measures.

## Electronic supplementary material

Below is the link to the electronic supplementary material.

Supplementary Material 1

## Abbreviations

ATS: American Thoracic Society
BMI: Body mass index
COPD: Chronic obstructive pulmonary disease
FEF: Forced expiratory flow
FEV1: Forced expiratory volume in the first second
FVC: Forced vital capacity
GOLD: Global Initiative for Chronic Obstructive Lung Disease
HsCRP: High-sensitivity C reactive protein
IL-6: Interleukin 6
PEF: Peak expiratory flow
SAA: Serum amyloid A
