# Supplementary material for: Assessment of ventilatory functions and associated inflammatory markers among workers in slaughterhouses
Source: Int Arch Occup Environ Health. 2024 Aug 16;97(8):891–900. doi: 10.1007/s00420-024-02094-8 (PMC11485019; doi:10.1007/s00420-024-02094-8)
Supplement: Supplementary file 1 — Supplementary Material 1 [file 420_2024_2094_MOESM1_ESM.docx]

**Table 6: Post hoc pairwise comparisons for different degrees of obstruction with other variables.**

|  | **Age** | **Years of employment** | **IL6(pg/mL)** | **hsCRP(mg/L)** |
| --- | --- | --- | --- | --- |
|  | **P value** | **P value** | **P value** | **P value** |
| **No obstruction-Moderate obstruction** | 0.039^*^ | 0.032^*^ | < 0.001^*^ | 0.001^*^ |
| **No obstruction-Severe & very severe obstruction** | 0.023^*^ | 0.037^*^ | < 0.001^*^ | < 0.001^*^ |
| **Moderate obstruction-Severe & very severe obstruction** | 0.682 | 0.867 | 0.123 | 0.181 |

IL-6 = interleukin 6; hsCRP = high-sensitivity C reactive protein. *p-value < 0.05 denotes statistical significance.


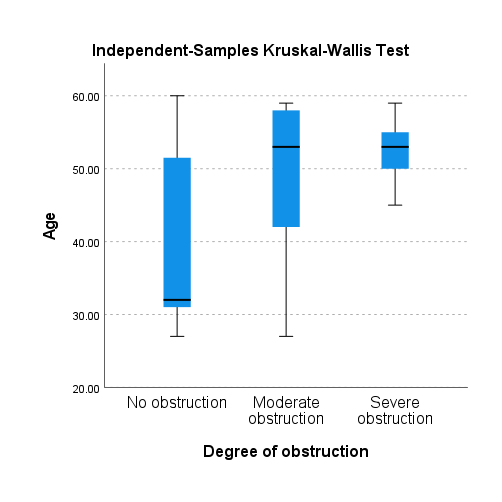

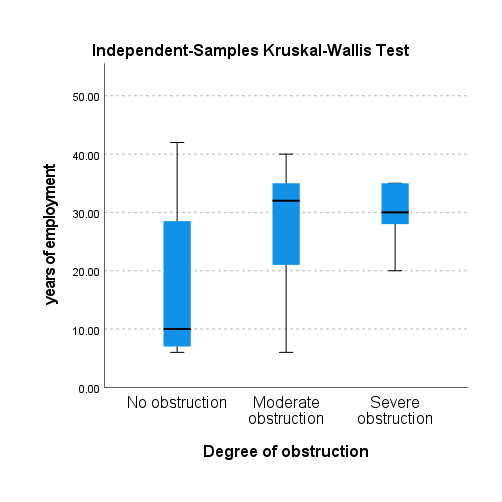


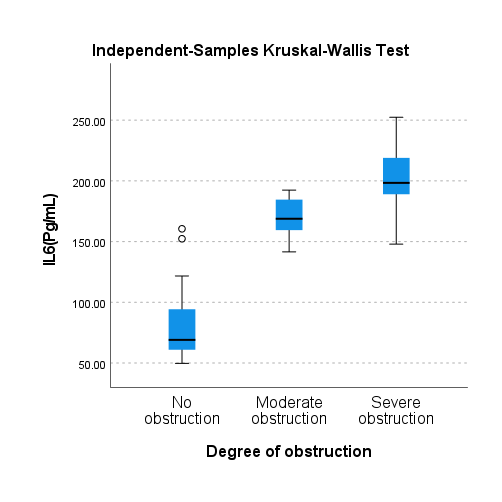

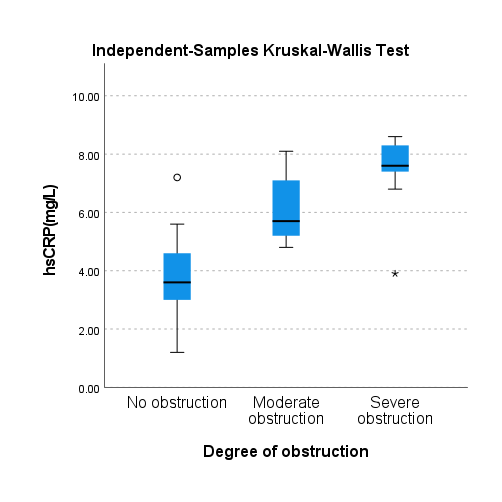


Figure (2): Kruskal Wallis test for pairwise comparison of different degrees of obstruction with different variables.
